# Genome-Wide Detection of Small Regulatory RNAs in Deep-Sea Bacterium *Shewanella piezotolerans* WP3

**DOI:** 10.3389/fmicb.2017.01093

**Published:** 2017-06-15

**Authors:** Muhammad Z. Nawaz, Huahua Jian, Ying He, Lei Xiong, Xiang Xiao, Fengping Wang

**Affiliations:** ^1^State Key Laboratory of Microbial Metabolism, School of Life Sciences and Biotechnology, Shanghai Jiao Tong UniversityShanghai, China; ^2^State Key Laboratory of Ocean Engineering, Shanghai Jiao Tong UniversityShanghai, China

**Keywords:** sRNA, *Shewanella piezoloterans* WP3, deep-sea, adaptation, gene regulation

## Abstract

*Shewanella* are one of the most abundant Proteobacteria in the deep-sea and are renowned for their versatile electron accepting capacities. The molecular mechanisms involved in their adaptation to diverse and extreme environments are not well understood. Small non-coding RNAs (sRNAs) are known for modulating the gene expression at transcriptional and posttranscriptional levels, subsequently playing a key role in microbial adaptation. To understand the potential roles of sRNAs in the adaptation of *Shewanella* toward deep-sea environments, here an *in silico* approach was utilized to detect the sRNAs in the genome of *Shewanella piezotolerans* WP3, a piezotolerant and psychrotolerant deep-sea iron reducing bacterium. After scanning 3673 sets of 5′ and 3′ UTRs of orthologous genes, 209 sRNA candidates were identified with high confidence in *S. piezotolerans* WP3. About 92% (193 out of 209) of these putative sRNAs belong to the class *trans*-encoded RNAs, suggesting that *trans*-regulatory RNAs are the dominant class of sRNAs in *S. piezotolerans* WP3. The remaining 16 *cis*-regulatory RNAs were validated through quantitative polymerase chain reaction. Five *cis*-sRNAs were further shown to act as cold regulated sRNAs. Our study provided additional evidence at the transcriptional level to decipher the microbial adaptation mechanisms to extreme environmental conditions.

## Tools Description:

QRNA, A tool used for the detection of the conserved RNA secondary structures, including both ncRNA genes and *cis*-regulatory RNA structures.RNAz, A program for predicting structurally conserved and thermodynamically stable RNA secondary structures in multiple sequence alignments.RNAalifold, A software used for the prediction of a consensus structure for a set of related RNAs.sRNAPredict3/SIPHT, sRNApredict3/SIPHT are recent versions of the sRNApredict suite that are used in the efficient prediction of sRNAs, with a high level of specificity. SIPHT is a compatible web version of sRNAPredict3 that searches approximately 1900 bacterial replicons from the NCBI database and predicts putative sRNA locations. sRNApredict3 is inclusive of sequence comparable options to look for conserved sRNAs.

sRNAscanner, The sRNAscanner is a computational tool used to detect the intergenic small RNA specific transcriptional units (TU) in the completely sequenced bacterial genome.

NAPP (Nucleic acid phylogenetic profiling), is a clustering method that efficiently identifies non-coding RNA (ncRNA) elements in a bacterial genome.

Rfam, The Rfam database is a collection of RNA families, each represented by multiple sequence alignments, consensus secondary structures and covariance models (CMs).

RNA Infernal, Infernal (“INFERence of RNA ALignment”) is a tool for searching DNA sequence databases for RNA structure and sequence similarities.

CLUSTALW, ClustalW is a general purpose DNA or protein multiple sequence alignment program for three or more sequences.

PAM, PAM (point accepted mutation) matrices are used as substitution matrices to score sequence alignments in CLUSTALW.

FastTree, is open-source software to infer approximately maximum-likelihood phylogenetic trees from alignments of nucleotide or protein sequences.

## Introduction

Until the early 1990s, non-coding DNA was assumed to be non-functional and referred to as Junk DNA (Pagel and Johnstone, 1992). From the past two decades, these non-coding sequences were shown to act as modulators and regulators of gene expression in response to environmental signals (Gottesman, 2005; Vogel and Wagner, 2007; Waters and Storz, 2009), and were usually small in size (50–500 nucleotide [nt]) (Vogel and Papenfort, 2006; Gottesman and Storz, 2011) called small non-coding RNA (sRNA) and were shown to involve in a variety of biological processes, including quorum sensing, bacterial virulence, iron homeostasis, stress responses and so on (Masse and Gottesman, 2002; Lenz et al., 2004; Thomason et al., 2012). The sRNA-encoding genes are widespread in bacterial genomes (Luban and Kihara, 2007) and sRNAs can regulate the gene expression both at transcriptional and post-transcriptional level (Gottesman and Storz, 2011). The sRNA that binds to target mRNA can act either as *cis* or *trans. Cis*-encoded sRNA is typically encoded adjacent to its regulatory target on the same strand as a riboswitch or on the opposite strand to an antisense sRNA, with a perfect base-pairing region between their transcripts (Wagner et al., 2002; Brantl, 2007). On the other hand, *trans*-encoded sRNA is separated from the target gene, where an imperfect base-pairing often occurs between their transcripts (Gottesman, 2002, 2005; He and Hannon, 2004; Aiba, 2007). *Trans*-regulatory sRNAs are believed to be involved in the regulation of several biological processes including iron homeostasis and quorum sensing (Gottesman and Storz, 2011) while most of the *cis*-regulatory RNAs are known to maintain the appropriate copy number of the mobile elements (Wagner et al., 2002; Brantl, 2007). To date, almost all the sRNA species identified are encoded in the intergenic regions (IGRs) (Koo et al., 2011). IGRs are under lower selection pressure when compared to the rest of genomic regions, allowing more room to mutate and evolve in response to various environmental stimuli.

Since the first discovery of sRNA using electrophoresis in *Escherichia coli* in 1967 (Hindley, 1967), recent advances in 2D-gel electrophoresis, Northern blotting, direct labeling and RNA sequencing, DNA microarray, shotgun sequencing, co-purification, and genomic SELEX (Systematic Evolution of Ligands by Exponential enrichment) have led us to enhanced identification of sRNAs (Vogel and Sharma, 2005; Vogel et al., 2005; Huttenhofer and Vogel, 2006; Altuvia, 2007; Livny and Waldor, 2007; Liu et al., 2009). Previous sRNA identification studies have mainly been carried out on extensively studied bacteria including *E. coli* (Hershberg et al., 2003), *Salmonella* (Hebrard et al., 2012), *Pseudomonas aeruginosa* (Livny et al., 2006), *Staphylococcus aureus* (Marchais et al., 2009), *Synechocystis* PCC 6803 (Voss et al., 2009), *Burkholderia pseudomallei* (Khoo et al., 2012), *B. cenocepacia* J2315 (Ramos et al., 2012), *Clostridium difficile* (Soutourina et al., 2013), *Brucella abortus* 2308 (Dong et al., 2014), and *Listeria monocytogenes* (Toledo-Arana et al., 2009). *E. coli* has been the most studied microbe in this context, almost 80 sRNAs, including 30 Hfq (host factor for Q2)-dependent ones, have been validated using various experimental approaches such as Northern blot and microarray (Altuvia, 2007; Waters and Storz, 2009). Hfq belongs to the large family of Sm and Sm-like proteins (a family of RNA binding proteins), that promotes the binding between sRNA and its target mRNA through conserved sequence motif (Møller et al., 2002). sRNAs such as *RyhB* (regulate iron homeostasis) have been characterized using microarrays. *OxyR* (oxidative stress induced RNA), and *CsrB* (carbon storage regulator) were discovered by co-purification with overproduced CsrA protein (Altuvia et al., 1997; Romeo, 1998). Growth phase dependent sRNA genes in *E. coli* and *S. aureus* were identified using DNA microarray along with comparative genome analysis (Wassarman et al., 2001; Pichon and Felden, 2005; Silvaggi et al., 2006). The sRNAs have also shown to play regulatory roles in response to fluctuating conditions, for instance, *RyhB* has shown to involve in regulation of tricarboxylic acid cycle under iron-limiting conditions, through modulating the *fur* (ferric uptake regulator) gene expression in *E. coli* (Modi et al., 2011; Salvail and Masse, 2012; Michaux et al., 2014).

However, experimental methods are tedious and time-consuming. Moreover, expressions of sRNAs are condition-dependent (Stubben et al., 2014), therefore, experimental verification of sRNAs are less effective and inconclusive (McHugh et al., 2014). As a result, many of the predicted sRNAs could not be verified using experimental methods (Gottesman and Storz, 2011). Alternatively, with the availability of *in silico* sRNA prediction algorithms, computational screening of sRNAs in a large/genomic scale becomes efficient and complementary to experimental approaches (Livny and Waldor, 2007; Khoo et al., 2012). Bio-computationally predicted sRNAs are subsequently validated through experiments (Argaman et al., 2001; Rivas and Eddy, 2001; Wassarman et al., 2001). Recently, computational tools based on different features, such as RNA secondary structures, thermodynamic stability, conservation of sequence and structure, transcriptional termination signals, and non-coding sequence clusters based on cross-genome conservation profiles (Vogel and Sharma, 2005; Lu et al., 2011), have greatly facilitated the efficient prediction of sRNAs in diverse bacterial species (Lu et al., 2011). Some of the widely used *de novo* search tools (**Table 1**) include QRNA (Rivas and Eddy, 2001), RNAz (Washietl and Hofacker, 2004), sRNAPredict3/SIPHT (Livny and Waldor, 2007), sRNAscanner (Aziz et al., 2010), RNAalifold (Bernhart et al., 2008), and NAPP (Nucleic acid phylogenetic profiling) (Ott et al., 2012). RNAz predicts evolutionarily conserved and thermodynamically stable RNA secondary structures in multiple sequence alignments, which is not only accurate as compared to other available tools (Xu et al., 2009) but also efficient as well (Washietl et al., 2005). Knowledge-based approaches, with homologs of identified sRNAs for profiling, can be used as complementary to *de novo* ones. RNA Infernal (Nawrocki and Eddy, 2007), one of the knowledge-based sRNA identification tool, together with a *de novo* tool RNAz were used in this study.

**Table 1:** Summary of computational discovery and validation of bacterial sRNAs.

Bacteria species	Computational Identification method	Experimental verification method	Number of sRNAs			Reference
				
			Predicted	Tested	Verified	
*Pseudomonas aeruginosa*	sRNAPredict2	Northern blot	2759	31	17	Livny et al., 2006
*Staphylococcus aureus*	NAPP	Northern blot	189	24	7	Marchais et al., 2009
*Synechocystis* PCC6803	RNAz	Northern blot	383	2	2	Voss et al., 2009
*Burkholderia pseudomallei*	SIPHT, sRNAScanner and RNA Infernal	RT-PCR	1306	15	8	Khoo et al., 2012
*Burkholderia cenocepacia* J2315	RNAalifold & TransTermHP	Northern blot	NA	24	24	Ramos et al., 2012
*Brucella abortus* 2308	SIPHT and NAPP	RT-PCR	129	20	7	Dong et al., 2014
*Shewanella piezotolerans* WP3	RNAz and RNA Infernal	RT-qPCR	209	16	15	This study


Not all predicted sRNAs can be verified by experimental techniques due to the fact that the functioning of sRNAs is condition-dependent. Due to the difficulty in experimental verification and characterization, only a small portion of computationally identified sRNA is subjected to testing. Up to date, a few studies on sRNA prediction and characterization have been conducted at a large scale, where at least over 100 sRNAs have been identified in each study (**Table 1**). In a study by Livny et al. (2006), in total 2759 sRNAs were predicted in *Pseudomonas aeruginosa*, but only 31 were tested and 17 were validated. According to Khoo et al. (2012), over 1300 sRNAs were identified in *Burkholderia pseudomallei*, 15 of which were tested and 8 were validated. Different computation tools have different sensitivities in generating sRNA candidates, varying from hundreds to thousands (**Table 1**), integrated use of tools provide a moderate and more accurate dataset for validation. The percentage of validated sRNAs relative to the total number of tested candidates can be as high as 100% in the case for *B. cenocepacia* J2315 (Ramos et al., 2012) and *Synechocystis* PCC6803 (Voss et al., 2009), and as low as 29% in *Staphylococcus aureus* (Marchais et al., 2009) (**Table 1**).

*Shewanella* are widely distributed aquatic organisms and one of the most abundant Proteobacteria in the deep sea, with the ability to grow on minimal medium and utilize a variety of compounds, such as iron, manganese, sulfite, oxygen, chromium, uranium, nitrate, fumarate, trimethylamine *N*-oxide (TMAO), and DMSO (Dimethyl sulfoxide), as terminal electron acceptors (Kato, 1999; Kato and Nogi, 2001). In addition, *Shewanella* has been shown to adapt to harsh and diverse environmental conditions with extreme temperature, pH, salinity, and pressure (Hau and Gralnick, 2007). *Shewanella piezotolerans* WP3 (hereafter referred as WP3), a piezotolerant and psychrotolerant Gram-negative gammaproteobacteria, was isolated from west Pacific deep-sea sediment at a depth of 1914 m (Wang et al., 2004). WP3 is considered as a good candidate for studying adaptation of *Shewanella* to deep-ocean (Xiao et al., 2007), as it grows in the pressure range 0.1–50 MPa with optimal growth at 20 MPa, and in the temperature range of 0–28°C with optimum growth temperature of 20°C (Xiao et al., 2007). WP3 has shown capable of adapting to a broad range of physical environmental conditions (Wang et al., 2008; Yang et al., 2013). Among the closely related species of WP3 from *Shewanella* genus, *S. oneidensis* MR-1 (referred as MR-1) is extensively studied and environmentally important species of the *Shewanella* genus because of its ability to use more than ten respiratory electron acceptors including nitrate, chromium, and uranium (Tiedje, 2002). *S. psychrophila* WP2 (referred as WP2) was isolated along with WP3 (Xiao et al., 2007), and shown to be a facultative anaerobic and psychrophilic, growing optimally at about 10–15°C. *S. violacea* DSS12 (referred as DSS12) is a piezo- and psychrophilic deep-sea bacterium which grows optimally at 8°C and pressure of 30 MPa (Kato et al., 1995). DSS12 and *S. benthica* KT99 (referred as KT99), two piezophiles (Lauro et al., 2013), are the only species belonging to *Shewanella* genus which is found to be present at the depth of more than 2,000 m in the ocean (Wang et al., 2004). Details of these 26 sequenced genomes of *Shewanella* genus used in this study are available in Supplementary Table S1. In the present study, we used an *in silico* approach to detect the sRNAs in the genome of *Shewanella piezotolerans* WP3 and predicted sRNAs were further characterized, validated through transcriptions and quantitative polymerase chain reaction (qPCR). Discovery of novel sRNAs in WP3 and studying their conservation patterns across deep-sea bacterial lineages will lead us to elucidate gene regulation and molecular mechanisms of bacterial adaptation to extreme deep-sea environmental conditions.

## Materials and Methods

### Intergenic Region Extraction and Orthologs Prediction

As bacterial regulatory motifs were found to be encoded in IGRs of the genome, each IGR dataset was generated by extracting sequences from both 5′ and 3′-untranslated regions (UTRs) of each gene in WP3 (described in details below), together with its orthologous IGRs in other reference genomes (Thomason and Storz, 2010). IGRs from 5′ and 3′-UTRs of the orthologous sets of genes were extracted and considered as orthologous sets of IGRs. Orthologous relationship of genes across all the reference genomes was predicted by using OrthoMCL (Li et al., 2003). Afterward, IGRs of each gene belonging to same orthologous groups (OG) in WP3 and reference genomes were retrieved as a set of orthologous IGR.

For each IGR dataset from 5′-end, up to 250 nt upstream (depending upon the length of IGR) and 20 nt downstream of the translation initiation sites of the corresponding genes in the positive direction were extracted and considered as one orthologous set of 5′-UTRs and subject to RNA secondary structure prediction. For the purpose of increased specificity and sensitivity, three overlapping windows, that is -250∼-100, -200∼-50 and -150∼+20 (1 corresponds to the translation start site, “-” referred to upstream, and “+” to downstream) were generated from each original IGR dataset and analyzed separately. For genes transcribing in the negative direction, 250 nt downstream and 20 nt upstream of translation initiation sites were extracted and divided into three overlapping windows in the same manner. Similarly, for the extraction of orthologous IGR datasets from 3′-end, up to 250 nt (depending upon the length of IGR) sequences downstream and 20 nt upstream of the translation initiation sites of genes in the positive direction were retrieved as one orthologous set and subject to RNA secondary structure prediction. This IGR region was divided into three overlapping windows that are -20∼150, 50–200, 100–250. For genes transcribing in the negative direction, 250 nt upstream and 20 nt downstream of translation initiation site were extracted and divided into three overlapping windows.

### sRNA Prediction by RNAz and RNA Infernal

We applied a comparative genomics-based approach for prediction of bacterial sRNA by integrating the RNA secondary structure prediction tool RNAz (Washietl et al., 2005) and RNA motif searching tool RNA-Infernal^1^. RNAz is widely used and comparatively reliable for *de novo* detection of structured non-coding RNAs from comparative genomics analysis (Washietl et al., 2005; Gruber et al., 2010). This program predicts structurally conserved and thermodynamically stable RNA secondary structures from multiple sequence alignments, with training data from multiple Rfam families and RNAalifold (Bernhart et al., 2008) for common RNA structure prediction. RNA Infernal is a homology-based tool that searches sequence databases for homologs of structural RNA sequences and then generates structure-based RNA sequence alignments. As stated above, IGRs were extracted from 5′ and 3′-UTRs of genes in target genome and its orthologous regions in reference genomes. Afterward, multiple sequence alignment for each IGR orthologous dataset was performed with CLUSTALW (Thompson et al., 1994) (using PAM substitution matrix). RNAz was then applied on multiple aligned sequences, with default parameters. Orthologous UTR sets that were predicted as RNA by RNAz were searched in Rfam database using the RNA motif searching software Infernal (Nawrocki and Eddy, 2007), where highly confident candidates were those, which predicted with an infernal score higher than 10 bit (CM score > 10).

### Conservation of Predicted sRNAs

We used the same approach to find the conservation or genome specificity of predicted *cis*-sRNAs in few closely related species of *Shewanella* genus and well-studied piezophilic and psychrophilic bacteria. We retrieved the 5′-UTR of a gene in WP3 and 5′-UTRs of orthologs of this gene in other species of *Shewanella* genus or other genera as a set of orthologous IGR. We applied RNAz on these orthologous sets of IGRs, positively predicted sets by RNAz were further subject to RNA Infernal. If the sequence in the set of orthologous IGR that belongs to 5′-UTR in WP3 and have infernal score higher than 10, it was declared as sRNA in WP3. Other sequences belonging to other species in the same set of orthologous IGR were considered as conserved if they also gave an infernal score higher than 10. These predicted sRNAs are conserved sRNAs because they are orthologous to each other as they were found at the 5′-UTR of the orthologous genes.

### Phylogenetic Tree Construction

**FIGURE 1:**
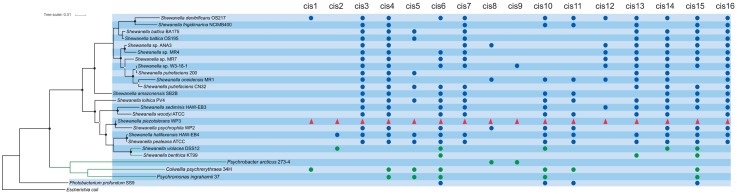
Phylogenetic relationships of the species used in this studies and conservation pattern of 16 identified sRNAs. 16S rRNA gene sequences based maximum-likelihood tree of species used in this studies shows their taxonomic relationship. Bootstrap values are based on 1000 replicates and are shown with white (≥80%) and black (≥90%) circles. sRNAs in WP3 are shown in red triangles, sRNAs in psychrophilic species are shown in green circles while sRNAs in other related species are indicated with blue circles.

Maximum-likelihood tree based on 16S rRNA gene sequences was constructed to represent the taxonomic relationships of the species used in this study and tree was used to demonstrate the conservation pattern of 16 identified *cis*-sRNAs across species used in present study. 16S rRNA gene sequences of all the representative species were aligned using CLUSTALW and tree was constructed with FastTree using default parameters. Bootstrap values are based on 1000 replicates and are shown with white (≥80%) and black (≥90%) circles (**Figure 1**).

### Culture Conditions and RNA Isolation

*Shewanella piezotolerans* WP3 was cultured in a modified marine medium 2216E (5 g/liter tryptone, 1 g/liter yeast extract, 0.1 g/liter FePO_4_, 34 g/liter NaCl) (Xiao et al., 2007). For aerobic cultivation, the single clone of WP3 strains were firstly inoculated into a 5 ml test tube, then the culture was diluted 1000-fold in the same medium with shaking (220 rpm) at 0.1 MPa (1 atm) and 20 and 4°C, respectively. For anaerobic cultivation, media was prepared without any electron acceptor under non-sterile condition. Prepared media was dispensed into serum bottles filled with O_2_-free nitrogen, and bottles were covered with stoppers. Metal seals were used to seal the caps and media was autoclaved by inserting a needle into the stopper. Needles were plucked off immediately after the autoclave was done. Then serum bottles were filled by a gassing manifold system after the media was cooled down (Balch and Wolfe, 1976). Nitrate solution (0.4 M, sodium nitrate) was filter sterilized in a vinyl anaerobic airlock chamber (Coy Laboratory Products Inc., Grass Lake, MI, United States) and added to the concentration needed. The serum bottles, stoppers, and metal seals were bought from Wheaton Science Products (Millville, NJ, United States). In order to incubate WP3 at high pressure, log phase cultures of WP3 cells at atmospheric pressure were grown and then diluted 1000-fold with the same medium for anaerobic cultivation. After that cells were transferred into sterile injection syringes and were placed inside the pressure vessels. The syringes were then incubated at a hydrostatic pressure of 20 MPa (200 atm) at 4°C. Pin closure pressure vessels were used in this study (Feiyu Petrochemical Instrument Equipment Inc., Nantong, China). Pressure was applied using a hand-operated pump with a quick-fit connector to the pressure vessel.

The growth of the WP3 strains was determined using turbidity measurements at 600 nm with a spectrophotometer (UV-2550, Shimadzu, Kyoto, Japan). The culture of WP3 was collected immediately when the cells reached mid-exponential phase (OD_600_≈0.8 and 0.2 for aerobic and anaerobic cultivation, respectively). The samples were centrifuged for 30 s at the maxim speed (16000 × *g*). The cells were immediately frozen in liquid nitrogen for subsequent RNA extraction.

TRI reagent-RNA/DNA/protein isolation kit (MRC, Cincinnati, OH, United States) was used to isolate the total RNA. After treating the RNA samples with DNase I at 37°C for 1 h, RNA was purified by using RNeasy Mini Kit (Qiagen, Hilden, Germany). The quality of RNA samples were determined by visualizing the nearly 2:1 ratio of 23S:16S ribosomal RNA by running a 1.0% TAE (Tris-Acitate-EDTA) agarose gel. The total RNA was treated with DNase I at 37°C for 1 h to remove DNA contamination and the purity was checked by PCR amplification with RNA as template. The quantity and integrity of RNA was evaluated with a UV spectrophotometer (Thermo Fisher, Waltham, MA, United States). In general, the ratio of 260 nm/280 nm > 2 and 260 nm/280 nm≈2.2 indicate the RNA is pure and could be used for the follow-up analysis. cDNA was synthesized from the purified RNA samples with the RevertAid First Strand cDNA Synthesis Kit (Fermentas, Glen Burnie, MD, United States).

### Microarray Analysis of Differentially Expressed Genes at Low Temperature

The expression profiles of genes in WP3 genome at low temperature (4°C compared to 25°C) were studied using microarray data (NCBI GEO dataset accession: GSE80668). A microarray that contained 95% of the total predicted genes of WP3 was designed and manufactured (CapitalBio, Beijing, China). Differentially expressed genes (DEGs) were the ones with a significant change (fold ≥ 2) in expression pattern. Microarray signals with *P*-values < 0.001 in the *F*-test were regarded as DEGs. All of the DEGs were confirmed with the Significance Analysis of Microarrays (SAM) software. Detailed descriptions of the microarray procedure for WP3 under cold temperature was described somewhere else (Jian et al., 2016).

### Real-Time qPCR

Primer Express software (ABI) was used to design the primer pairs for the selected genes for real-time qPCR (qPCR). PCR cycling was conducted using 7500 System SDS software (ABI, Foster City, CA, United States) in reaction mixtures with total volumes of 20 μl containing 1× SYBR Green I Universal PCR Master Mix (ABI, Foster City, CA, United States), 0.5 μM each primer, 1 μl cDNA template. The amount of target was normalized to that of the reference gene *swp2079*, whose expression remains constant under various conditions relative to the calibrator (The transcription levels of the genes at 20°C and 0.1 MPa were set as 1) (Li et al., 2006). qPCR assays were performed in triplicate for each sample, and a mean value and standard deviation were calculated for the relative RNA expression levels. Primers designed for qPCR are shown in Supplementary Table S2.

## Results

sRNAs are considered to be evolutionarily conserved in their secondary structures among the closely related species (Livny and Waldor, 2007). Here, we used a comparative genomics-based approach for genome-wide screening of sRNAs. At present, various species of *Shewanella* genus from various environments have been sequenced. In this comparative genomics-based approach, 26 representatives sequenced genomes of *Shewanella* genus were selected based on niche and phylogenetic relatedness and being well-studied and were used as reference genomes, including one partial sequenced genome (*Shewanella benthica* KT99), being closely related to WP3. We have also included a genome sequence of *Shewanella psychrophila* WP2 isolated previously along with WP3 (Wang et al., 2004). This unpublished completely sequenced genome was also studied and discussed here in this study. In addition, we also investigated the distribution pattern of the identified *cis*-regulatory RNAs in WP3, across 10 bacterial species for piezophilic and psychrophilic adaptation features, including four selected *Shewanella* species (MR-1, WP2, DSS12, and KT99), three piezophilic model bacteria [*Photobacterium profundum* SS9 (Vezzi et al., 2005), *Marinitoga piezophila* KA3 (Lucas et al., 2012), *Desulfovibrio piezophilus* C1TLV30 (Khelaifia et al., 2011), referred as SS9, KA3, and C1TLV30], and three well studied psychrophilic bacteria (*Colwellia psychrerythraea* 34H, *Psychromonas ingrahamii* 37, *Psychrobacter arcticus* 273-4, referred as Cp34H, Pi37, and Pa273-4).

### sRNA Prediction in WP3 and Other *Shewanella* Species

Using an integrated approach 209 RNA motifs in WP3 were predicted as reliable sRNA candidates. For comparative analysis of occurrence of sRNAs and determining the conservation of sRNAs, genomes of closely related species including MR-1, WP2, DSS12, and KT99 were scanned for sRNAs identification, using the same approach (**Table 2**). From total sets of 5′ and 3′-UTRs in WP3 and other four *Shewanella* species, about 3 to 8% were predicted as sRNA candidates. As *cis*-regulatory RNAs are often located in the 5′-UTR of the mRNA (Xu et al., 2009), predicted sRNAs from 5′-UTRs are considered as *cis*-regulatory and sRNAs from 3′-UTRs as *trans*-regulatory RNAs (Papenfort et al., 2015). *Cis*-regulatory RNAs are mainly known to maintain the appropriate copy number of the mobile elements (Wagner et al., 2002; Brantl, 2007), while *trans*-regulatory RNAs are associated with almost every global response in bacteria (Gottesman and Storz, 2011). Because of their ubiquitous roles, *trans*-acting sRNAs are extensively studied and well characterized in Gram-negative bacteria (Man et al., 2011). However, compared to *trans*-sRNAs, *cis*-sRNAs are much less understood. In the present study, the proportion of predicted *cis*-encoded sRNA was lower than that of *trans-*encoded sRNAs in WP3, with a ratio for *cis-* and *trans-*sRNA 1:12, and the roles of all the 16 predicted *cis*-sRNA (referred as cis1-16 herein) in WP3 will be discussed here in this paper.

**Table 2:** Number of datasets created, sRNAs predicted by RNAz and sRNAs further confirmed through RNA infernal tool with infernal score 10 or higher (High confidence sRNAs) in WP3 and four related species of *Shewanella* genus.

Species names	Number of datasets created			Positively predicted by RNAz			Putative sRNAs having infernal score > 10 (high confidence sRNAs)		
			
	5′-UTR Datasets	3′-UTR Datasets	Total Datasets	From 5′-UTR Dataset	From 3′-UTR Dataset	From total Dataset	From 5′-UTR Dataset	From 3′-UTR Dataset	Total identified sRNA
*Shewanella piezotolerans* WP3	2276	1397	3673	172	561	733	16	184	200
*Shewanella oneidensis* MR-1	1991	1268	3259	131	537	668	27	190	217
*Shewanella psychrophila* WP2	2325	1574	3899	171	633	804	24	157	181
*Shewanella violacea* DSS12	1939	1409	3348	147	561	708	57	211	268
*Shewanella benthica* KT99	2016	1402	3418	159	577	736	15	142	157


### Conservation of Identified *Cis*-sRNA

The distribution pattern of the 16 identified *cis*-regulatory RNAs in WP3 across selected piezophilic and psychrophilic bacterial species (details in Materials and Methods) was displayed in **Figure 1**. Seven sRNAs (cis2, cis3, cis7, cis12, cis13, cis14, and cis16) were found limited to *Shewanella* genus, so they are more likely to be *Shewanella* specific sRNAs. Nine out of the 16 predicted *cis*-regulatory RNAs have been found universally conserved in at least half of all the reference genomes presented in this study (**Figure 1**). Only one piezophilic species *Photobacterium profundum* SS9 shared four conserved *cis*-encoded sRNA with WP3, while in the rest of two piezophiles (*Marinitoga piezophila* KA3 and *Desulfovibrio piezophilus* C1TLV30) no common sRNAs was identified. One of these four conserved *cis*-sRNAs in *Photobacterium profundum* SS9 (conserve with cis15 in WP3) was already identified in a study by Campanaro et al. (2012) (locus tag = PBPRA0551b). All the four psychrophilic species used in the present study shared conserved *cis*-sRNAs with WP3, where five sRNA (cis4, cis5, cis6, cis10, and cis15) were conserved in at least half of the all psychrophilic species used in this study (**Figure 1**).

### Function Inference from Genome Annotation

All 16 *cis*-regulatory RNAs were annotated against the known regulatory motifs in Rfam database (**Table 3**). Among the five *cis-*sRNAs conserved in psychrophilic bacteria, cis4 was annotated as *asX2*, an sRNA which appears to function in virulence (Schmidtke et al., 2012). cis5 was annotated as *rimP* with a possible role in the regulation of NusA protein (a protein which functions in 30S ribosomal subunit maturation) (Nord et al., 2009; Naville and Gautheret, 2010), cis6 as *nsiR* with a function in cell differentiation (Muro-Pastor, 2014). cis10 was with the best hit as Atu_C9, with reported roles in growth (Wilms et al., 2012), while cis15 was with the best hit to STnc180 with unknown function (Sittka et al., 2008). According to the genome annotation of WP3, the 5′ genes of both cis4 and cis5 is anthranilate synthase component I (TrpE), an enzyme involved in the conversion of chorismate to anthranilate, while the 5′ gene of cis6 is 2-isopropylmalate synthase, which plays an important role in the biosynthesis of l-leucine and pyruvate metabolism (Webster and Gross, 1965; Cole et al., 1973). The 5′ gene for cis10 is ATP phosphoribosyltransferase (biosynthesis of histidine), and for cis15 is aspartate kinase (phosphorylation of the amino acid aspartate). Locations of identified *cis*-sRNAs and their neighborhood genes in the genome of WP3 are shown in **Supplementary Figure S1**.

**Table 3:** Rfam best hits of identified *cis*-encoded sRNAs in WP3 and changes in expression profiles of 5′-associated genes of identified *cis*-sRNAs under cold temperature conditions.

Sr. #	Identified *cis*-sRNA	Rfam Hits of sRNAs			Putative target genes of *cis*-sRNAs	
			
		Best hit	Best hitting organism	Function	5′-associated genes of *cis*-sRNA in WP3	Expression of associated genes under cold temperature
1	cis1	isrJ (RF01393)	*Salmonella typhimurium*	Virulence	Peptidase M48, Ste24p	Up-regulated
2	cis2	isrK (RF01394)	*Salmonella typhimurium*	Adaptation in low oxygen and low Mg level	Carbamoyl-phosphate synthase, small subunit	Down-regulated
3	cis3	rimP (RF01770)	Gammaproteobacteria	Ribosome maturation factor	Anthranilate synthase component I, TrpE	Up-regulated
4	cis4	asX2 (RF02236)	*Xanthomonas campestris*	Virulence	Anthranilate synthase component I, TrpE	Up-regulated
5	cis5	rimP (RF01770)	Gammaproteobacteria	Ribosome maturation factor	Anthranilate synthase component I, TrpE	Up-regulated
6	cis6	NsiR1 (RF02399)	*Anabaena* sp. strain PCC 7120	Cell differentiation	2-isopropylmalate synthase	Up-regulated
7	cis7	NsiR1 (RF02399)	*Anabaena* sp. strain PCC 7120	Cell differentiation	2-isopropylmalate synthase	Up-regulated
8	cis8	BjrC68 (RF02353)	*Bradyrhizobiaceae*	Unknown	MSHA pilin protein MshB	Down-regulated
9	cis9	BjrC68 (RF02353)	*Bradyrhizobiaceae*	Unknown	MSHA pilin protein MshB	Down-regulated
10	cis10	Atu_C9 (RF02503)	*Rhizobiales*	Growth	ATP phosphoribosyltransferase	Up-regulated
11	cis11	ar15 (RF02345)	*Alphaproteobacteria*	Unknown	ATP phosphoribosyltransferase	Up-regulated
12	cis12	CsrC (RF00084)	*Escherichia coli*	Regulator of carbon storage regulatory (Csr) system	3-oxoacyl-(acyl-carrier-protein) synthase II	Down-regulated
13	cis13	Atu_C9 (RF02503)	*Rhizobiales*	Growth	PpiC-type peptidyl-prolyl *cis-trans* isomerase	Down-regulated
14	cis14	STnc250 (RF01409)	*Enterobacteriaceae*	Stress response regulator	Trigger factor	Down-regulated
15	cis15	STnc180 (RF02079)	*Enterobacteriaceae*	Stress response regulator	Aspartate kinase	Up-regulated
16	cis16	STnc100 (RF02076)	*Gammaproteobacteria*	Stress response regulator	Chorismate mutase, gammaproteobacteria	Up-regulated


### Validation Using Microarray Data and qPCR

**FIGURE 2:**
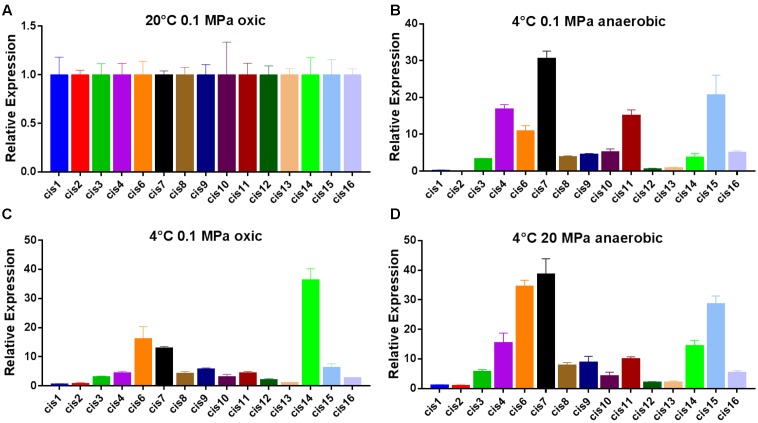
Expression of identified *cis*-sRNAs under different conditions determined by qPCR. **(A)** Relative expression of identified *cis*-sRNAs at 20°C, 0.1 MPa, and under oxic conditions. **(B)** Relative expression of identified *cis*-sRNAs at 4°C, 0.1 MPa, and under oxic conditions. **(C)** Relative expression of identified *cis*-sRNAs at 4°C, 0.1 MPa, and under anaerobic conditions. **(D)** Relative expression of identified *cis*-sRNAs at 4°C, 20 MPa, and under anaerobic conditions.

As *cis*-regulatory RNAs are responsible for regulating the gene expression of their 5′-associated genes, gene expression of identified *cis*-sRNAs and their associated genes at optimum and cold temperature conditions were determined using RT-qPCR and microarray data, respectively. Five *cis*-sRNA (cis4, cis5, cis6, cis10, and cis15) were appeared conserve in psychrophilic species and were considered to have a role in cold adaptation and later qPCR analysis showed their increased expression under all cold conditions (**Figure 2**), therefore regarded as cold-regulated sRNAs. Interestingly, 5′-genes of all the five cold-regulated sRNAs were shown to be up-regulated under cold temperature conditions (**Table 3**). As *cis*-regulatory RNAs in bacteria are typically located at the 5′-end of their target genes and are involved in regulating the gene expression of their 5′-associated gene (Weinberg et al., 2010). Therefore, it is inferred that these predicted *cis*-sRNAs present at the 5′-end of the gene are contributing toward regulating the expression of their 5′-associated gene. All the 16 *cis*-sRNAs were also validated through RT-qPCR except cis5, a possible explanation of this exception can be that it might be expressed under some other specific conditions. qPCR results showed that 9 *cis*-sRNAs (cis4, cis6-11, cis14-15), including four cold regulators were present with an increase in transcription under all three low-temperature conditions (**Figure 2**). As these cold-regulated sRNAs were found with increased transcription under cold temperature conditions consistent with their target regulatory genes, it shows that they have positive regulatory effect over their 5′-associated genes, i.e., in response to cold temperature condition, increase in the sRNA transcription takes place which promotes the transcription (up-regulation) of their target regulatory genes.

## Discussion

In present study, we extracted the IGR both from 5′ and 3′-UTR of the orthologous genes in WP3 and reference genomes and applied combination of RNAz and RNA Infernal tool to predict sRNAs in our target bacteria WP3 and four related species of *Shewanella* genus, i.e., *S. oneidensis* MR-1, *S. psychrophila* WP2, *S. violacea* DSS12, and *S. benthica* KT99. Positively predicted sRNAs based on thermodynamic stability by RNAz were subjected to motif searching tool RNA infernal. sRNA candidates present at the 5′-UTRs which were positively predicted by RNAz and giving an infernal score higher than 10 bit were called as “confident candidates” (Xu et al., 2009), and were subsequently selected for verification through RT-qPCR. In addition to CM bit Score (Covariance model), trusted cutoffs (TC) bit scores thresholds were also used in the model to evaluate sRNAs candidates. As TC thresholds are generally considered to be the score of lowest-scoring known true positive, about half of putative *cis*-sRNAs validated through RT-qPCR were missed, when TC bit score thresholds (–cut_tc parameter for using CM’s trusted cutoff thresholds) was used. In this study, we mainly focused on the less understood type of sRNAs (*cis*-sRNA) and reported that *cis*-regulatory RNAs might have a role in adaptation to extreme conditions. We also explored the conservation of *cis*-sRNAs in other reference species and their conservation pattern demonstrates that most of the sRNAs tend to remain to conserve within the genus. Conservation pattern of *cis*-sRNAs showed that five of them were conserved not only in psychrophilic species from *Shewanella* genus but also in species belonging to other genera. Furthermore, we compared the expression of their 5′-associated genes at an optimum and cold temperature and surprisingly, expression of all the 5′-associated genes were found with an increase in expression at low temperature. Regulated genes of all the five conserved sRNAs in psychrophiles are involved in the biosynthesis and metabolism of amino acids, secondary metabolites, and antibiotics, suggesting that bacterium tends to increase the metabolism of amino acids, secondary metabolites, and antibiotics at cold temperature.

As most studied class of sRNAs, *trans*-regulatory RNAs were found to be associated with almost every global response in bacteria and were reported as an abundantly existing class of regulatory RNAs in bacterial genomes (Gottesman and Storz, 2011). Likewise, about 92% (193 out of 209) of the sRNAs identified in this study, belonging to the class *trans*-encoded RNAs, which reflects that *trans*-regulatory RNAs are the dominant class of regulatory RNAs in *S. piezotolerans* WP3. We screened the orthologs of *trans*-encoded sRNAs in Rfam database where most of the *trans*-sRNAs shared homology with known *trans*-regulatory RNAs in *E. coli*, *S. enterica*, *Staphylococcus aureus*, *Cyanobacterium*, *Xanthomonas campestris*, *Vibrio cholera*, *Listeria monocytogenes*, and *Rhizobiales* species performing a variety of biological roles. Detailed information about the *trans*-sRNAs in the genome of *S. piezotolerans* WP3 and their orthologs in Rfam are available in Supplementary Table S3.

In short, we identified sRNAs in a piezo- and psychrotolerant, iron reducing, deep-sea bacterium, *Shewanella piezotolerans* WP3 and four closely related species of WP3. In total, we identified 209, 217, 181, 268, and 157 sRNAs in WP3, MR-1, WP2, DSS12, and KT99, respectively. Out of 209 predicted sRNAs in WP3, 16 are the *cis*-sRNAs (cis1-16) and were further characterized, validated through qPCR. Seven of this 16 *cis*-sRNAs (cis2, cis3, cis7, cis12, cis13, cis14, and cis16) were found as *Shewanella-*specific while rest of nine *cis*-regulatory RNAs were shown to be universal in at least half of all the genomes presented in this study. Five *cis*-sRNA (cis4, cis5, cis6, cis10, and cis15) were found as regulators commonly present in most of psychrophilic species used in this study. Expression analysis of 16 *cis*-sRNAs and their target genes demonstrated significant change under low-temperature conditions. Although the present study does not provide insights into the functioning of all the identified sRNAs (including *cis* and *trans*) in *Shewanella piezotolerans* WP3, further analysis is required to reveal the possible roles of these sRNAs in WP3. However, our study also provides evidence that not only *trans-* but *cis*-sRNAs could also play their roles in adaptation to extreme conditions. It should be very useful to explore the conserved sRNAs in extremophiles adapted to diverse environmental conditions.

## Author Contributions

FW, XX, YH, and MN designed the experiment and analysis. MN designed the pipeline and performed the computational analysis. LX conducted experiments. MN, HJ, YH and FW wrote the manuscript, in consultation with all other authors.

## Conflict of Interest Statement

The authors declare that the research was conducted in the absence of any commercial or financial relationships that could be construed as a potential conflict of interest.

## Footnotes

1 http://eddylab.org/infernal/

## Supplementary Material

**FIGURE S1 |** Identified *cis*-sRNAs in WP3 genomes and their flanking genes. Neighborhood genes are shown in green wedges and sRNAs are shown in blue colored round corner rectangles. Genes without any labels are the hypothetical proteins.
