# Supplementary figures and images for: Genome-Wide Detection of Small Regulatory RNAs in Deep-Sea Bacterium Shewanella piezotolerans WP3
Source: Front Microbiol. 2017 Jun 15;8:1093. doi: 10.3389/fmicb.2017.01093 (PMC5471319; doi:10.3389/fmicb.2017.01093)

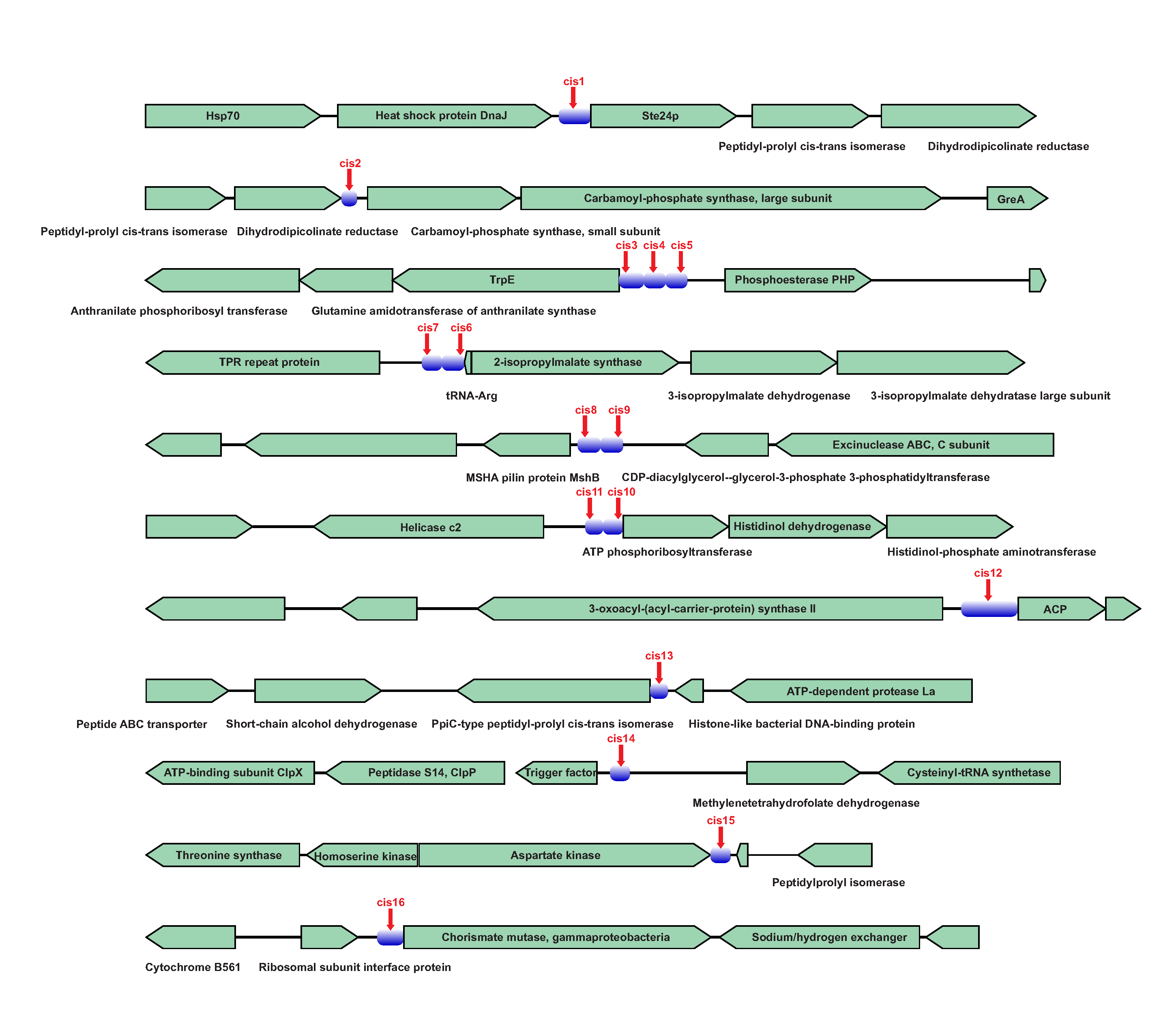

Supplement: Supplementary file 1 [file Image_1.TIFF]
