# Supplementary material for: Genome-Wide Detection of Small Regulatory RNAs in Deep-Sea Bacterium Shewanella piezotolerans WP3
Source: Front Microbiol. 2017 Jun 15;8:1093. doi: 10.3389/fmicb.2017.01093 (PMC5471319; doi:10.3389/fmicb.2017.01093)
Supplement: Supplementary file 2 [file Table_1.DOC]

**Genome-wide detection of small regulatory RNAs in deep-sea bacterium *Shewanella piezotolerans* WP3**

Muhammad Zohaib Nawaz^1,2^, Huahua Jian^1^, Ying He^1,2^, Lei Xiong^1^, Xiang Xiao^1,2^, Fengping Wang^1,2*^

^1^State Key Laboratory of Microbial Metabolism, School of Life Sciences and Biotechnology, Shanghai Jiao Tong University, Shanghai, 200240, People’s Republic of China.

^2^State Key Laboratory of Ocean Engineering, Shanghai Jiao Tong University, Shanghai, 200240, People’s Republic of China

*Corresponding author’s email: [fengpingw@sjtu.edu.cn](mailto:fengpingw@sjtu.edu.cn).

**SUPPLEMENTARY DATA**

**Supplementary Table S1: 26 genomes of *Shewanella* genus used in present study**

| **Sr.#** | **Species/Strain Name** | **Site of Isolation** | **Isolation site Characteristics** | **Genome Size (bp)** |
| --- | --- | --- | --- | --- |
| 1 | *Shewanella amazonesis* SB2B | Amapa River Brazil | Sediment:suboxic conditions: 1m | 4306142 |
| 2 | *Shewanella* sp. *ANA*3 | Woods Hole, Massachusetts, USA | Brackish water:arsenic-treated wooden pier | 5396476 |
| 3 | *Shewanella baltica* BA175 | Baltic sea | Oxic-Anoxic interface of Baltic sea: 120 m | 5150660 |
| 4 | *Shewanella baltica* OS117 | Baltic sea | chemocline : 130 m | 5242964 |
| 5 | *Shewanella baltica* OS155 | Baltic sea | Sea water: oxic zone:2ml per liter of oxygen: 90 m | 5127376 |
| 6 | *Shewanella baltica* OS185 | Baltic sea | Sea water;oxic-anoxic interface: 120 m | 5229686 |
| 7 | *Shewanella baltica* OS195 | Baltic sea | Sea water;anoxic zone: 140 m | 5347283 |
| 8 | *Shewanella baltica* OS223 | Baltic sea | Sea water;oxic-anoxic interface: 120 m | 5145902 |
| 9 | *Shewanella baltica* OS678 | Baltic sea | Low oxygen zone: just above the chemocline: 110 m | 5288069 |
| 10 | *Shewanella benthica* KT99 | Abyssal South Pacific Ocean | Tonga-Kermadec trench at a depth of 9,856 m | Partial seq |
| 11 | *Shewanella denitrificans* OS217 | Baltic sea | Sea water;oxic-anoxic interface: 120 m | 4545906 |
| 12 | *Shewanella frigidimarina* NCIMB 400 | Coast of Aberdeen, UK | Sea water: North sea | 4845257 |
| 13 | *Shewanella halifaxensis* HAW EB4 | Halifax Harbor, Nova Scotia, Canada | Sediments: munitions dumping area: 215 m | 5226917 |
| 14 | *Shewanella loihica* PV4 | Hawaiian Sea mount, US | Iron-rich mat: hydrothermal vent: 1325 m | 4602594 |
| 15 | *Shewanella* sp. MR4 | Black sea | Sea-water:oxic zone:16^o^C: 5m | 4706287 |
| 16 | *Shewanella* sp. MR7 | Black sea | Sea-water:anoxic zone: high NO_3_ :60 m | 4792610 |
| 17 | *Shewanella oneidensis* MR1 | Lake Oneida, NY, USA | Sediment: anaerobic:MN (IV) reduction | 4969811 |
| 18 | *Shewanella pealeana* ATCC 700345 | Atlantic squid | accessory nidamental gland of the squid | 5174581 |
| 19 | Shewanella piezotolerans WP3 | West Pacific site | Sediment: under 1914 m of water | 5396476 |
| 20 | *Shewanella psychrophila* WP2 | West Pacific site | Sediment: under 1914 m of water | 6353472 |
| 21 | *Shewanella putrefaciens* 200 | Alberta, Canada | Crude-oil pipeline | 4840251 |
| 22 | *Shewanella putrefaciens* CN32 | Albuquerque, New Mexico, USA | Sub surface:Shale Sandstone: 250 m | 4659220 |
| 23 | *Shewanella sediminis* HAW-EB3 | Halifax Harbor, Nova Scotia, Canada | Sediment: 50 nautical miles from shore | 5517674 |
| 24 | *Shewanella violacea* DSS12 | Ryuki Trench, Philippine Sea | Sediment: 5110 m | 4962103 |
| 25 | *Shewanella* sp. *W3*-18-1 | Washington coast, Pacific Ocean | Marine sediment: under 997 m of oxic | 4708380 |
| 26 | *Shewanella woodyi* ATCC | Alborean Sea | detritus at a depth of 370 m in seawater | 5935403 |

**Supplementary Table S2: Pair of primers designed for RT-qPCR**

| **Primer** | **Sequence (5’-3’)** | **Base pairs** |
| --- | --- | --- |
| 1F | CCTTCTGCGATTCCCACTGA | 20 |
| 1R | AAAAGCCCCGCAATTGC | 17 |
| 2F | GTACAGTTCCACTATAGACGAGTA | 24 |
| 2R | TTGACAAATTCCGCCGATT | 19 |
| 3F | GCGATATCTTTTACTGCCATGCTA | 24 |
| 3R | GCAGCAGGCTCTTGATTTTTAAG | 23 |
| 4F | TTGTGCGGGCTTTTTTGTTT | 21 |
| 4R | AGCGGGTGGTGTGAAATTCT | 20 |
| 5F | CGCTGTGGTTTAGATAC | 17 |
| 5R | GGGTGGTGTGAAATTC | 16 |
| 6F | GATATAGTGCTACACGCTTA | 20 |
| 6R | CGTTCAGTTATGCTTTCAG | 19 |
| 7F | TGAAGCGTACAAGCACATTACTCA | 24 |
| 7R | TATCCTCCGCGCAGTGACT | 19 |
| 8F | CCAACTTTCGTTGAGGCTTAATG | 23 |
| 8R | CCGGCCTCGGGTACCA | 16 |
| 9F | GTTGTCACGAAATCAGTAGGTAATTGA | 27 |
| 9R | GGGTACCAAATATTCTCGCAAGA | 23 |
| 10F | CTGCCGAAGGACTGATT | 17 |
| 10R | AATTCCTTCCAGGGGTTTT | 19 |
| 11F | AACACTATCCATCATCATCACCATATG | 27 |
| 11R | CAACCGCCAGAAGGAATCAG | 20 |
| 12F | GCGAGGAGAATATTGCTTTAAGACTT | 26 |
| 12R | GCCGCCTGTTTTTGTTTCTG | 20 |
| 13F | CGCATCACTCGTTGGAT | 17 |
| 13R | GGCAATGGCTAAGTATTTACT | 21 |
| 14F | GAGCCTCAAAGCATGATTTAC | 21 |
| 14R | GTCACCCCACTCATTCTG | 18 |
| 15F | GCGGGCTTGATATTTAACTTAT | 22 |
| 15R | GATTATTACAACCACCACGATT | 22 |
| 16F | GGCGGAATTTCGGTGTAAAA | 20 |
| 16R | CCCGTGTGGGAGGCTTT | 17 |

**Supplementary Table S3: *Trans*-regulatory RNAs in *Shewanella piezotolerans*** WP3 and their orthologs in Rfam database

| **Sr. #** | **sRNA Name** | **sRNA coordinates** | **Orthologs in Rfam** | **Description of orthologs** | **Infernal Score** |
| --- | --- | --- | --- | --- | --- |
|  | trans1 | 27449-27598 | STnc250 | STnc250 Hfq binding RNA | 29 |
|  | trans2 | 27399-27548 | STnc250 | STnc250 Hfq binding RNA | 39 |
|  | trans3 | 27349-27498 | MicX | MicX *Vibrio cholerae* sRNA | 21 |
|  | trans4 | 115207-115356 | SpF51_sRNA | *Streptococcus* sRNA SpF51 | 14 |
|  | trans5 | 194658-194807 | NsiR1 | Nitrogen stress-induced RNA 1 | 18 |
|  | trans6 | 196444-196593 | tracrRNA | *Trans*-activating crRNA | 13 |
|  | trans7 | 393255-393404 | Atu_C8 | *Rhizobiales* sRNA Atu_C8 | 11 |
|  | trans8 | 416331-416480 | MicA | MicA sRNA (also known as SraD) | 12 |
|  | trans9 | 463357-463506 | rli40 | *Listeria* sRNA rli40 | 15 |
|  | trans10 | 480970-481119 | STnc40 | *Salmonella enterica* sRNA STnc40 | 18 |
|  | trans11 | 493900-494049 | GcvB | Hfq binding sRNA | 12 |
|  | trans12 | 499932-500081 | Histone3 | Histone 3'-UTR stem-loop | 25 |
|  | trans13 | 603016-603165 | sar | sar RNA | 14 |
|  | trans14 | 602966-603115 | sar | sar RNA | 13 |
|  | trans15 | 603236-603385 | sau-19 | *Staphylococcus* sRNA sau-19 | 25 |
|  | trans16 | 603286-603435 | L10_leader | Ribosomal protein L10 leader | 22 |
|  | trans17 | 697302-697451 | OrzO-P | OrzO-P RNA antitoxin family | 20 |
|  | trans18 | 703006-703155 | Leu_leader | Leucine operon leader | 32 |
|  | trans19 | 705328-705477 | isrK | Hfq binding sRNA | 18 |
|  | trans20 | 709397-709546 | greA | *Enterobacteria* greA leader | 16 |
|  | trans21 | 714405-714554 | RsaJ | RNA *Staph. aureus* A | 31 |
|  | trans22 | 793788-793937 | STnc430 | *Enterobacterial* sRNA STnc430 | 12 |
|  | trans23 | 797897-798046 | Hairpin | Hairpin ribozyme | 14 |
|  | trans24 | 811159-811308 | Betaproteobacteria_toxic_sRNA | Toxic Small RNA (tsRNA) | 10 |
|  | trans25 | 838998-839147 | CsrB | CsrB/RsmB RNA family | 12 |
|  | trans26 | 903496-903645 | STnc250 | STnc250 Hfq binding RNA | 17 |
|  | trans27 | 918178-918327 | sau-6072 | *Staphylococcus* sRNA sau-6072 | 12 |
|  | trans28 | 918128-918277 | sau-6072 | *Staphylococcus* sRNA sau-6072 | 11 |
|  | trans29 | 1112665-1112814 | STnc50 | *Salmonella enterica* sRNA STnc40 | 16 |
|  | trans30 | 1114352-1114501 | Phe_leader | Phenylalanine leader peptide | 21 |
|  | trans31 | 1145763-1145912 | STnc420 | *Salmonella enterica* sRNA | 10 |
|  | trans32 | 1148114-1148263 | MicX | MicX *Vibrio cholerae* sRNA | 22 |
|  | trans33 | 1169789-1169938 | Rhino_CRE | Human rhinovirus internal CRE | 12 |
|  | trans34 | 1177581-1177730 | STnc100 | Gammaproteobacterial sRNA STnc100 | 18 |
|  | trans35 | 1186147-1186296 | STnc410 | Enterobacterial sRNA STnc410 | 16 |
|  | trans36 | 1197358-1197507 | Trp_leader | Tryptophan operon leader | 37 |
|  | trans37 | 1197408-1197557 | CsrC | CsrC RNA family | 35 |
|  | trans38 | 1202790-1202939 | isrJ | Hfq binding sRNA | 18 |
|  | trans39 | 1205344-1205493 | isrK | Hfq binding sRNA | 26 |
|  | trans40 | 1209942-1210091 | sX11 | *Proteobacterial* sRNA sX11 | 25 |
|  | trans41 | 1214691-1214840 | STnc350 | *Salmonella enterica* sRNA STnc350 | 44 |
|  | trans42 | 1214641-1214790 | BjrC1505 | *Alphaproteobacterial* sRNA BjrC1505 | 35 |
|  | trans43 | 1233352-1233501 | P24 | *Pseudomonas* sRNA P24 | 13 |
|  | trans44 | 1236185-1236334 | Leu_leader | Leucine operon leader | 12 |
|  | trans45 | 1281472-1281621 | Atu_C8 | *Rhizobiales* sRNA Atu_C8 | 12 |
|  | trans46 | 1287868-1288017 | SNORD52 | Small nucleolar RNA SNORD52 | 11 |
|  | trans47 | 1384761-1384910 | TarB | *Vibrio* ToxT activated RNA TarB | 16 |
|  | trans48 | 1391827-1391976 | ctRNA_pND324 | ctRNA | 15 |
|  | trans49 | 1392499-1392648 | GlmZ_SraJ | GlmZ RNA activator of glmS mRNA | 10 |
|  | trans50 | 1405757-1405906 | sau-63 | *Staphylococcus* sRNA sau-63 | 36 |
|  | trans51 | 1426115-1426264 | isrJ | Hfq binding sRNA | 10 |
|  | trans52 | 1454179-1454328 | STnc380 | *Enterobacterial* sRNA STnc380 | 11 |
|  | trans53 | 1491135-1491284 | snoR128 | Small nucleolar RNA snoR128 | 14 |
|  | trans54 | 1491136-1491285 | snoR128 | Small nucleolar RNA snoR128 | 12 |
|  | trans55 | 1505197-1505346 | Phe_leader | Phenylalanine leader peptide | 13 |
|  | trans56 | 1509253-1509402 | Leu_leader | Leucine operon leader | 21 |
|  | trans57 | 1510541-1510690 | STnc380 | *Enterobacterial* sRNA STnc380 | 11 |
|  | trans58 | 1740836-1740985 | NrrF | NrrF RNA | 16 |
|  | trans59 | 1744727-1744876 | GcvB | Hfq binding sRNA | 28 |
|  | trans60 | 1752985-1753134 | Thr_leader | Threonine operon leader | 29 |
|  | trans61 | 1754650-1754799 | Atu_C8 | *Rhizobiales* sRNA Atu_C8 | 16 |
|  | trans62 | 1767782-1767931 | Trp_leader | Tryptophan operon leader | 11 |
|  | trans63 | 1767832-1767981 | NsiR1 | Nitrogen stress-induced RNA 1 | 13 |
|  | trans64 | 1781045-1781194 | L10_leader | Ribosomal protein L10 leader | 11 |
|  | trans65 | 1867324-1867473 | Atu_C9 | *Rhizobiales* sRNA Atu_C9 | 20 |
|  | trans66 | 1903217-1903366 | rpsL_ricks | *Rickettsia* rpsL leader | 20 |
|  | trans67 | 1911822-1911971 | Leu_leader | Leucine operon leader | 13 |
|  | trans68 | 1926604-1926753 | Thr_leader | Threonine operon leader | 23 |
|  | trans69 | 1999368-1999517 | STnc320 | sRNA STnc320 | 15 |
|  | trans70 | 2001569-2001718 | STnc420 | *Salmonella enterica* sRNA STnc420 | 33 |
|  | trans71 | 2037211-2037360 | MIR159 | microRNA MIR159 | 16 |
|  | trans72 | 2043865-2044014 | sraA | sraA | 20 |
|  | trans73 | 2060251-2060400 | rimP | proteobacterial rimP leader | 10 |
|  | trans74 | 2077828-2077977 | P26 | *Pseudomonas* sRNA P26 | 10 |
|  | trans75 | 2086400-2086549 | Pseudomonas sRNA P26 | *Salmonella enterica* sRNA STnc40 | 44 |
|  | trans76 | 2105653-2105802 | Phe_leader | Phenylalanine leader peptide | 10 |
|  | trans77 | 2140470-2140619 | Atu_C9 | *Rhizobiales* sRNA Atu_C9 | 20 |
|  | trans78 | 2147908-2148057 | Trp_leader | Tryptophan operon leader | 11 |
|  | trans79 | 2295118-2295267 | rimP | γ-*proteobacterial* rimP leader | 20 |
|  | trans80 | 2308140-2308289 | Entero_OriR | *Enteroviral* 3' UTR element | 11 |
|  | trans81 | 2334224-2334373 | MicX | MicX *Vibrio cholerae* sRNA | 24 |
|  | trans82 | 2422645-2422794 | His_leader | Histidine operon leader | 18 |
|  | trans83 | 2425237-2425386 | GlmZ_SraJ | GlmZ RNA activator of glmS mRNA | 24 |
|  | trans84 | 2470273-2470422 | rsmX | rsmX | 30 |
|  | trans85 | 2497926-2498075 | STnc180 | *Enterobacterial* sRNA STnc180 | 22 |
|  | trans86 | 2543149-2543298 | STnc40 | *Salmonella enterica* sRNA STnc40 | 35 |
|  | trans87 | 2548161-2548310 | NsiR1 | Nitrogen stress-induced RNA 1 | 18 |
|  | trans88 | 2589288-2589437 | Atu_C8 | *Rhizobiales* sRNA Atu_C8 | 19 |
|  | trans89 | 2589360-2589509 | STnc50 | *Salmonella enterica* sRNA STnc40 | 12 |
|  | trans90 | 2613850-2613999 | MIR405 | microRNA MIR405 | 10 |
|  | trans91 | 2620670-2620819 | Spot_42 | Spot 42 (spf ) RNA | 24 |
|  | trans92 | 2643091-2643240 | Trp_leader | Tryptophan operon leader | 21 |
|  | trans93 | 2663423-2663572 | rnk_pseudo | *Pseudomonas* rnk leader | 19 |
|  | trans94 | 2669322-2669471 | tRNA | Transfer RNA | 14 |
|  | trans95 | 2712683-2712832 | mir-282 | microRNA mir-282 | 22 |
|  | trans96 | 2721043-2721192 | STnc320 | sRNA STnc320 | 17 |
|  | trans97 | 2729540-2729689 | Thr_leader | Threonine operon leader | 23 |
|  | trans98 | 2731903-2732052 | Phe_leader | Phenylalanine leader peptide | 34 |
|  | trans99 | 2739247-2739396 | STnc40 | *Salmonella enterica* sRNA STnc40 | 60 |
|  | trans100 | 2758435-2758584 | Thr_leader | Threonine operon leader | 28 |
|  | trans101 | 2758485-2758634 | isrJ | Hfq binding sRNA | 29 |
|  | trans102 | 2768048-2768197 | SCARNA2 | Small Cajal body-specific RNA 2 | 24 |
|  | trans103 | 2810158-2810307 | SpF51_sRNA | *Streptococcus* sRNA SpF51 | 13 |
|  | trans104 | 2879384-2879533 | mir-318 | microRNA mir-318 | 29 |
|  | trans105 | 2909635-2909784 | mir-553 | microRNA mir-553 | 38 |
|  | trans106 | 2946090-2946239 | Phe_leader | Phenylalanine leader peptide | 17 |
|  | trans107 | 2960845-2960994 | L20_leader | Ribosomal protein L20 leader | 23 |
|  | trans108 | 3082548-3082697 | SpF39_sRNA | *Streptococcus* sRNA SpF39 | 21 |
|  | trans109 | 3092620-3092769 | Leu_leader | Leucine operon leader | 31 |
|  | trans110 | 3177697-3177846 | SurA | SurA sRNA | 14 |
|  | trans111 | 3194781-3194930 | STnc130 | *Enterobacteria* sRNA STnc130 | 27 |
|  | trans112 | 3196220-3196369 | CsrC | CsrC RNA family | 27 |
|  | trans113 | 3208273-3208422 | Atu_C9 | *Rhizobiales* sRNA Atu_C9 | 28 |
|  | trans114 | 3213213-3213362 | STnc130 | *Enterobacteria* sRNA STnc130 | 32 |
|  | trans115 | 3260373-3260522 | Leu_leader | Leucine operon leader | 22 |
|  | trans116 | 3275885-3276034 | snR80 | Small nucleolar snR80 | 10 |
|  | trans117 | 3291749-3291898 | L20_leader | Ribosomal protein L20 leader | 20 |
|  | trans118 | 3305543-3305692 | STnc40 | *Salmonella enterica* sRNA STnc40 | 25 |
|  | trans119 | 3307185-3307334 | PK-repBA | PK of regulatory region of repBA | 19 |
|  | trans120 | 3313293-3313442 | whalefall-1 | Whalefall-1 RNA | 13 |
|  | trans121 | 3368519-3368668 | Thr_leader | Threonine operon leader | 17 |
|  | trans122 | 3414431-3414580 | BjrC174 | *Bradyrhizobiaceae* sRNA BjrC174 | 17 |
|  | trans123 | 3416452-3416601 | Atu_C9 | *Rhizobiales* sRNA Atu_C9 | 29 |
|  | trans124 | 3431544-3431693 | Leu_leader | Leucine operon leader | 17 |
|  | trans125 | 3479946-3480095 | Thr_leader | Threonine operon leader | 18 |
|  | trans126 | 3526662-3526811 | isrK | Hfq binding sRNA | 12 |
|  | trans127 | 3558973-3559122 | Betaproteobacteria_toxic_sRNA | Toxic Small RNA (tsRNA) | 30 |
|  | trans128 | 3594625-3594774 | rimP | γ-*proteobacterial* rimP leader | 25 |
|  | trans129 | 3643257-3643406 | STnc50 | *Salmonella enterica* sRNA STnc40 | 10 |
|  | trans130 | 3664739-3664888 | STnc390 | *Salmonella* sRNA STnc390 | 13 |
|  | trans131 | 3672637-3672786 | RybB | RybB RNA | 18 |
|  | trans132 | 3731186-3731335 | STnc370 | *Enterobacterial* sRNA STnc370 | 19 |
|  | trans133 | 3824443-3824592 | MicX | MicX *Vibrio cholerae* sRNA | 57 |
|  | trans134 | 3824393-3824542 | SpF25_sRNA | *Streptococcus* sRNA SpF25 | 40 |
|  | trans135 | 3837834-3837983 | mir-48 | Micro RNA mir-48 | 16 |
|  | trans136 | 3844792-3844941 | mir-577 | Micro RNA mir-577 | 12 |
|  | trans137 | 3948193-3948342 | FsrA | FsrA | 10 |
|  | trans138 | 3948093-3948242 | STnc370 | *Enterobacterial* sRNA STnc370 | 31 |
|  | trans139 | 3948143-3948292 | sraA | sraA | 10 |
|  | trans140 | 3955417-3955566 | Atu_C9 | *Rhizobiales* sRNA Atu_C9 | 16 |
|  | trans141 | 3959736-3959885 | RsmY | RsmY RNA family | 29 |
|  | trans142 | 3971344-3971493 | Atu_C9 | *Rhizobiales* sRNA Atu_C9 | 19 |
|  | trans143 | 3978553-3978702 | STnc420 | *Salmonella enterica* sRNA STnc420 | 10 |
|  | trans144 | 3981119-3981268 | P9 | *Pseudomonas* sRNA P9 | 11 |
|  | trans145 | 4011970-4012119 | Thr_leader | Threonine operon leader | 24 |
|  | trans146 | 4116506-4116655 | mir-77 | microRNA mir-77 | 13 |
|  | trans147 | 4122232-4122381 | sroD | sroD RNA | 12 |
|  | trans148 | 4189138-4189287 | sraA | sraA | 15 |
|  | trans149 | 4231719-4231868 | RsaOG | RNA *S.aureus* Orsay G | 14 |
|  | trans150 | 4235335-4235484 | rimP | γ-*proteobacterial* rimP leader | 17 |
|  | trans151 | 4256747-4256896 | ar15 | *Alphaproteobacterial* ar15 | 13 |
|  | trans152 | 4265592-4265741 | Qrr | Qrr RNA | 13 |
|  | trans153 | 4332046-4332195 | isrK | Hfq binding sRNA | 27 |
|  | trans154 | 4350034-4350183 | Trp_leader | Tryptophan operon leader | 12 |
|  | trans155 | 4384730-4384879 | Thr_leader | Threonine operon leader | 18 |
|  | trans156 | 4407023-4407172 | SpF25_sRNA | Streptococcus sRNA SpF25 | 19 |
|  | trans157 | 4421587-4421736 | Qrr | Qrr RNA | 22 |
|  | trans158 | 4439009-4439158 | STnc50 | *Salmonella enterica* sRNA STnc40 | 15 |
|  | trans159 | 4446180-4446329 | ST7-AS1_2 | ST7 antisense RNA 1 conserved | 10 |
|  | trans160 | 4447114-4447263 | STnc370 | *Enterobacterial* sRNA STnc370 | 27 |
|  | trans161 | 4505464-4505613 | L20_leader | Ribosomal protein L20 leader | 11 |
|  | trans162 | 4528334-4528483 | STnc430 | *Enterobacterial* sRNA STnc430 | 11 |
|  | trans163 | 4550895-4551044 | Atu_C9 | *Rhizobiales* sRNA Atu_C9 | 32 |
|  | trans164 | 4555154-4555303 | rli40 | *Listeria* sRNA rli40 | 17 |
|  | trans165 | 4555104-4555253 | rli40 | *Listeria* sRNA rli40 | 14 |
|  | trans166 | 4594633-4594782 | Phe_leader | Phenylalanine leader peptide | 31 |
|  | trans167 | 4618980-4619129 | STnc400 | *Salmonella enterica* sRNA STnc400 | 14 |
|  | trans168 | 4623482-4623631 | STnc320 | sRNA STnc320 | 21 |
|  | trans169 | 4681573-4681722 | RNAI | RNAI | 17 |
|  | trans170 | 4681523-4681672 | sroH | sroH RNA | 10 |
|  | trans171 | 4781091-4781240 | L20_leader | Ribosomal protein L20 leader | 11 |
|  | trans172 | 4946309-4946458 | RsaF | RNA *Staph. aureus* F | 16 |
|  | trans173 | 4960158-4960307 | Phe_leader | Phenylalanine leader peptide | 25 |
|  | trans174 | 4960169-4960318 | Phe_leader | Phenylalanine leader peptide | 31 |
|  | trans175 | 4968293-4968442 | SpF51_sRNA | *Streptococcus* sRNA SpF51 | 22 |
|  | trans176 | 4977700-4977849 | SpF51_sRNA | *Streptococcus* sRNA SpF51 | 26 |
|  | trans177 | 5025666-5025815 | STnc370 | *Enterobacterial* sRNA STnc370 | 33 |
|  | trans178 | 5032360-5032509 | sX11 | *Proteobacterial* sRNA sX11 | 17 |
|  | trans179 | 5072032-5072181 | snoR35 | Small nucleolar RNA snoR35 | 11 |
|  | trans180 | 5150044-5150193 | sraA | sraA | 32 |
|  | trans181 | 5155578-5155727 | LhrC | *Listeria* Hfq binding LhrC | 12 |
|  | trans182 | 5229912-5230061 | SurA | SurA sRNA | 18 |
|  | trans183 | 5246612-5246761 | Phe_leader | Phenylalanine leader peptide | 17 |
|  | trans184 | 5296727-5296876 | L17DE | L17 ribosomal protein downstream element | 10 |
|  | trans185 | 5297216-5297365 | mir-302 | microRNA mir-302 | 11 |
|  | trans186 | 5297316-5297465 | STnc100 | *Gammaproteobacterial* sRNA STnc100 | 27 |
|  | trans187 | 5297266-5297415 | frnS | Fumarate/nitrate reductase regulator sRNA | 10 |
|  | trans188 | 5298930-5299079 | STnc540 | *Enterobacterial* sRNA STnc540 | 19 |
|  | trans189 | 5303602-5303751 | rsmX | rsmX | 30 |
|  | trans190 | 5376820-5376969 | STnc430 | *Enterobacterial* sRNA STnc430 | 27 |
|  | trans191 | 5379656-5379805 | suhB | suhB | 15 |
|  | trans192 | 5382105-5382254 | MicX | MicX *Vibrio cholerae* sRNA | 22 |
|  | trans193 | 5384521-5384670 | Thr_leader | Threonine operon leader | 24 |
